# Registered nurses' experiences of working in the intensive care unit during the COVID‐19 pandemic

**DOI:** 10.1111/nicc.12649

**Published:** 2021-05-10

**Authors:** Lina Bergman, Ann‐Charlotte Falk, Axel Wolf, Ing‐Marie Larsson

**Affiliations:** ^1^ Department of Neurobiology, Care Sciences and Society Karolinska Institute Stockholm Sweden; ^2^ Department for Health Promoting Science Sophiahemmet University Stockholm Sweden; ^3^ Institute of Health and Care Sciences, Sahlgrenska Academy, University of Gothenburg Gothenburg Sweden; ^4^ Department of Surgical Sciences, Anaesthesiology and Intensive Care Uppsala University Uppsala Sweden

**Keywords:** COVID‐19, nursing, occupational health, pandemic, qualitative research

## Abstract

**Background:**

During the pandemic, increased numbers of patients requiring intensive care unit (ICU) admission required an increase in ICU capacity, including ICU staffing with competence to care for critically ill patients. Consequently, nurses from acute care areas were called in to staff the ICU along with experienced intensive care nurses.

**Aims and objectives:**

To describe Swedish registered nurses' experiences of caring for patients with COVID‐19 in ICUs during the pandemic.

**Design:**

Mixed method survey design.

**Methods:**

An online questionnaire was distributed through social media to registered nurses who had been working in the ICU during the COVID‐19 outbreak. Data were collected for 1 week (May 2020) and analysed using content analysis and descriptive statistics.

**Results:**

Of the 282 nurses who participated, the majority were ICU nurses (n = 151; 54%). Half of the nurses specialized in ICU reported that they were responsible for the ICU care of three or more patients during the pandemic (n = 75; 50%). Among non‐intensive care nurses, only 19% received introduction to the COVID‐19 ICU (n = 26). The analysis of data regarding nurses' experiences resulted in three categories: *tumbling into chaos*, *diminished nursing care*, and *transition into pandemic ICU care*. Participants described how patient safety and care quality were compromised, and that nursing care was severely deprioritized during the pandemic. The situation of not being able to provide nursing care resulted in ethical stress. Furthermore, an increased workload and worsened work environment affected nurses' health and well‐being.

**Conclusions:**

The findings from the present study indicate that nurses perceived that patient safety and quality of care were compromised during the pandemic. This resulted in ethical stress among nurses, which may have affected their physical and psychosocial well‐being.

**Relevance to clinical practice:**

The COVID‐19 pandemic had a severe impact on nurses' work environment, which could result in burnout and staff turnover.

1

**What is known about this topic**

- Nurse staffing levels and competence are associated with favourable patient outcomes.
- Increasing the ICU capacity during a pandemic implies increased ICU staffing, yet there is a general lack of registered nurses with competence to care for seriously ill patients.
- Frontline health care workers during pandemics might experience post‐traumatic stress and psychological distress.

**What this paper adds**

- Nurses described that patient safety in the ICU was compromised during the initial phase of the pandemic.
- Nurses transferred to work in the ICU required training and proper introduction and often lacked support.
- Nursing care was severely deprioritized during the pandemic, which was attributed to lack of time, resources, and competence needed.
- The notion of being unable to provide crucial elements of nursing care resulted in ethical stress among nurses.

## 2 INTRODUCTION

At the end of 2019, a novel coronavirus, SARS‐CoV2 was identified, which on 11th March 2020 was characterized as a pandemic and started to spread throughout the world.^1^ In Sweden, the pandemic peaked during March to May 2020, resulting in higher intensive care unit (ICU) needs. Before the pandemic, Sweden had a total of 526 ICU beds, but only 207 beds provided a full spectrum of monitoring and life support technologies (level III units).^2^ From a European perspective, Sweden had the second lowest number of intensive care beds (5.8 beds per 100 000 inhabitants) in Europe (range 4.2‐29.2 beds) even before COVID‐19.^3^ While increasing ICU capacity implies increasing hospital beds, equipment, and pharmaceuticals, it is a real challenge to increase ICU staffing with competence to care for seriously ill patients. National data from Swedish health care providers have shown a lack of both registered and specialized nurses throughout Sweden, especially in emergency care.^4^

## 3 BACKGROUND

COVID‐19 is associated with severe conditions that require intensive care in approximately 5% of cases^5^; the most common reason for intensive care is acute respiratory distress syndrome and the need for mechanical ventilation.^6^ According to Swedish intensive care registry data, there was an increase in ICU care days from 20 566 days in 2019 to 40 214 days in 2020 (April‐May).^7^ To meet the need for staffing, health care personnel from non‐ICU areas were transferred to the ICU setting. The outcomes in patient care as well as future resilience for treating COVID‐19 patients depend heavily on the competence, stamina, and well‐being of nurses who treat patients during the pandemic. The average ICU stay increased to 5.25 days (April‐May 2020) compared with 2.65 days (April‐May 2019). During the same period, the overall ICU mortality, which includes patients with and without COVID‐19, increased from 7.45% (2019) to 9.4% in 2020.^7^ A recently published meta‐analysis showed that the pooled prevalence of ICU mortality among confirmed COVID‐19 patients was 39%.^8^ However, because national data presented in this study included ICU patients with and without COVID‐19, the mortality rates between Sweden and other countries are difficult to compare. Mental health outcomes among frontline health care workers during COVID‐19 have previously been described and include post‐traumatic stress symptoms as well as other psychological distress symptoms.^9^ However, little is known about how nurses experienced working in the ICU during the acute phase of the COVID‐19 pandemic. This knowledge is vital to enhance quality of care, patient safety, and staffs' work environment during future pandemics.

## 4 AIM

The present study aimed to describe Swedish registered nurses' experiences of caring for patients with COVID‐19 in ICUs during the pandemic.

## 5 METHODS

A mixed method survey was developed based on the checklist for reporting results of internet surveys.^10^ This study was conducted and reported adhering to the consolidated criteria for reporting qualitative research guidelines.^11^

### 5.1 Setting and sample

The study was conducted in Sweden with nurses who were working in the ICU during the COVID‐19 outbreak. In Sweden, registered nurses specializing in intensive or anaesthetic care undertake additional specialist training, including a 1‐year master's degree. In Swedish ICUs, the nurse‐to‐patient ratio is normally 1:1‐2. The speciality is multidisciplinary, and the team caring for critically ill patients consists of specialist nurses, nurse assistants, specialist physicians, and physiotherapists. This study used a convenience sampling approach.^12^ Potential participants were specialized in either ICU or anaesthesia. Participants were invited through the Facebook page of the Swedish Association for Anaesthesia and Critical Care Nurses. The Facebook page is public and might therefore have followers from other specialities as well.

### 5.2 Questionnaire

An online questionnaire was developed by an expert group consisting of intensive care and anaesthesia specialist nurses (n = 4). Before distribution, the questionnaire was reviewed by a reference group of intensive care and anaesthesia care nurses (n = 4) and minor revisions of items were made. As the primary purpose of the present questionnaire was to collect experience of nurses working in the frontline of the pandemic, no further reliability or validity testing was performed. The questionnaire consisted of 13 multiple‐choice questions, including questions about participants' specialist training, years of clinical experience, workplace, number of patients per shift, and introduction and training with regard to COVID‐19 patients. It also included three open‐ended questions: (a) share your own reflection of working in the ICU during COVID‐19, (b) give an example of how nursing care has been affected, (c) can you tell us about a significant event that you have experienced during the pandemic?

### 5.3 Data collection

Data were collected from 10 to 17 May 2020. Information about the study, with a link to the survey, was presented on the Facebook page of the Swedish Association for Anaesthesia and Critical Care nurses, comprising over 1389 followers (10 May 2020).

### 5.4 Data analysis

Quantitative data were processed by Excel and presented using descriptive statistics (frequencies and percentages). Qualitative data were analysed using manifest content analysis, with an inductive approach.^13^ Answers from the open‐ended questions were read and assimilated into the data to achieve an overall perspective. Thereafter, data were condensed and coded into categories. Categories were then reviewed and revised, findings were summarized, and extracts selected. The first step of the analysis (i.e., coding and abstraction of preliminary categories) was primarily performed by one of the researchers (L.B.), with all of the researchers (A.‐C.F., A.W., and I.‐M.L.) involved in refining and revising categories and finalizing the results. The research team consisted of four nurses specialized in either intensive or anaesthetic care with experience of working in the ICU during the COVID‐19 pandemic. Further, they were all members of the professional organization that initiated and performed the survey.

### 5.5 Ethical considerations

The respondents received information concerning the study's aim, including that data and anonymized quotes would be published in both national and international literature. The questionnaire was anonymous, and by answering the questionnaire, respondents agreed to the terms for publishing. This procedure corresponds to the World Medical Association's ethical principles that no ethical approval was needed when the respondents agree to participate by answering the posted questions.^14^ The study did not need to seek ethical approval, as the Swedish Ethical Review Act (2003:406) does not include studies that do not handle sensitive data and patient data.

## 6 RESULTS

A total of 282 nurses participated, of whom the majority were ICU nurses (n = 151; 54%). All regions in Sweden were represented, and 52% of the participants worked at ICUs designated only for COVID‐19 patients (Table 1). Most non‐ICU nurses (n = 94, 72%) reported caring for one or two patients and 50% (n = 75) of the ICU nurses reported caring for three or more patients.

**TABLE 1:** Demographics of the respondents (N = 282)

	n (%)
Education	
Specialist in intensive care	136 (48)
Specialist in anaesthesia	106 (38)
Specialist in both anaesthesia and intensive care	15 (5)
Other (theatre nurses or non‐specialized nurses)	25 (9)
Country region	
Northern Sweden	28 (10)
Central Sweden	106 (38)
Southern Sweden	138 (49)
Missing	10 (3)
Years of experience	
<1	23 (8)
2‐5	86 (31)
6‐10	53 (19)
>10	119 (42)
Missing	1 (0.4)
I work at my regular workplace	
Yes	109 (39)
No	71 (25)
Partly	102 (36)
I work at a designated COVID‐19 ICU	
Yes	144 (52)
No	24 (8)
Partly	114 (40)

Nurses responded to questions about support, education, and training. Approximately half of the participants received sufficient support by senior ICU nurses in caring for patients during the pandemic. One‐third (n = 46, 34%) of non‐ICU nurses and half (n = 75, 50%) of ICU nurses felt confident in their COVID‐19 practice. Among non‐ICU nurses, only 19% (n = 26) received an introduction when starting their shifts in COVID‐19 ICUs.

### 6.1 Qualitative analysis

A total of 179 participants answered at least one of the three open‐ended questions. We analysed the answers from these questions and three categories were identified: (a) tumbling into chaos, (b) diminished nursing care, and (c) transition to pandemic ICU care.

#### 6.1.1 Tumbling into chaos

At the beginning of the COVID‐19 pandemic, nurses described their work as “being in a warzone.” Participating nurses reported finding themselves in situations that they described as “chaotic,” “surrealistic,” and “unreal.” Caring for patients with COVID‐19 in the ICU posed many challenges. Many of the nurses reported that patients were severely ill and unstable, and some described that they were the sickest patients they ever cared for.

In the beginning, you were overwhelmed when you came to work. […]. I will always remember those first weeks. It felt like it was a warzone, and I just have to make sure that my patients survive during my shift. This is for real and it is bad. (Participant 137)

During the COVID‐19 pandemic, nurses described how prerequisites for providing ICU care shifted. This included organizational changes, such as more patients per nurse (i.e., a decreased nurse‐to‐patient ratio) and environmental changes when, for example, post‐operative units or theatres were quickly redesigned to become ICU wards to care for patients in need of mechanical ventilation. Furthermore, there was a lack of medical supplies such as ventilators, commonly used sedation, and other protective equipment such as face shields and masks. As a consequence, nurses had to quickly learn how to use old or different mechanical ventilators, and they had to adapt to a situation when they no longer could rely on having access to supplies and resources.

The quality of nursing care (and the ICU care in general) is lower than usual. Access to medical equipment and supplies, time, and staff resources are severely reduced. (Participant 113)

Participating nurses stated that patient safety and quality of care were severely compromised during the pandemic. Several respondents described how they had to side‐step existing safety routines. They also had to constantly prioritize among the patients as the resources were limited and many expressed concerns about both short‐ and long‐term consequences. Commonly, medical equipment was re‐used, and participants described how minimum standards for ICU care could not be upheld. Nurses also expressed that there was a lack of support from ICU management. Thus, many decisions about prioritizing nursing and medical interventions had to be done by the nurses themselves.

The high standard of nursing care that we have in the ICU has decreased. A lot of the duties that we normally do can't be performed because we don't have the supplies needed and there is a shortage of staff. (Participant 20)

We have sidestepped from our normal routines and now we do things that we normally never would have accepted […]. Patient safety is totally affected. (Participant 93)

The nurses transferred to the ICU during the pandemic described how they received little or no introduction to their new workplace (normally a designated COVID‐19 ICU). Many expressed how they lacked competence and experience and thus often felt insecure or alone. Some described how they had been promised to work closely together with experienced intensive care nurses to receive assistance if needed; however, they were often solely responsible for two or more ICU patients. In addition, experienced intensive care nurses described how their workload increased as they constantly had to introduce and help new colleagues.

There is a great responsibility for us with experience and competence in intensive care. We have to lead the work, support and teach our new colleagues, and at the same time be responsible for many patients besides those we care for ourselves. (Participant 137)

I am an Anaesthetic nurse. Beforehand, I was told that I would not be solely responsible for the patients in the ICU, rather that I would help the ICU nurses. On my first day at a COVID‐ICU, I found myself responsible for two ICU patients in ventilators that I am not used to. No introduction, no one who showed me anything, nothing. I had to figure things out on my own. To my help, I had two OR nurses who normally worked in another hospital. (Participant 202)

Moreover, the respondents stated that they did not have a choice other than to work in an ICU setting during the pandemic. This was particularly evident among those who had been transferred to the ICU.

We (nurse anaesthetists) were forced to work at the ICU during COVID‐19. […]. It is stressful and we are worried; at the same time, we cannot do anything about the situation. (Participant 181)

The respondents further stressed how the increased workload and worsened work environment caused them physical and psychological stress.

It has been extremely demanding to work in personal protective equipment, and besides there is the psychological aspect of the risk that you might get infected and sick yourself, even die. (Participant 162)

Thus, working during the COVID‐19 pandemic affected nurses' health and well‐being. Some described how they were constantly thinking about work. Furthermore, symptoms of stress, such as nightmares, were reported among the respondents. Many also emphasized that they were so exhausted and tired that they did not have energy to do anything else.

I feel worried. It is physically demanding and physiologically even worse. This is the worst thing I have experienced during my career. (Participant 147)

#### 6.1.2 Diminished nursing care

During COVID‐19, the respondents expressed that the ICU had turned into something comparable to an industrial assembly line, where patients lined up in rows and were given the same kind of treatment. Most of the participating nurses stated that nursing care had been severely deprioritized during the pandemic. They described how they did not have time to provide nursing interventions to the patients because of lack of time, resources, competence, and medical/technical equipment. This included proactive interventions such as mouth care, weaning from mechanical ventilation, pressure injury prevention, mobilization, and screening and prevention of ICU delirium.

We do not have the time to do all the things that we usually do, such as oral care or changing the patients posture in bed. Relatives, who normally are a resource, cannot even visit the patient until he or she is dying. (Participant 70)

The fact that we are not as many nurses influences the quality of nursing. This affects our ability to work according to evidence‐based practice. Additionally, the ICU environment distresses the patients. The possibility of personal integrity is severely limited. (Participant 71)

Many of the respondents expressed concerns regarding being unable to communicate with the patients, especially when they were awake and had been extubated, because of the obstructions posed by the personal protective equipment. Furthermore, during the pandemic, relatives were not allowed to visit the ICU, or the visiting hours were restricted, and nurses seldom had any contact with patient's relatives. Thus, little was known about each patient's life and history. Many of the participating nurses described how this affected them and caused concerns. As a result, they described how patients treated in the ICU became dehumanized.

I found out where one of my patients worked. It became more like it usually is, a little, but more personal. The rest of the patients were almost like the same one. It was just a social security number; we did not know who we were treating. This was very dehumanising. Like someone said, it feels like we are just caring for a body. (Participant 23)

End‐of‐life care in the ICU is particularly challenging. Sometimes relatives were allowed to visit when the patient was dying, but several participants described situations when this was not possible. In those cases, the nurses had to act as a patient relative proxy to provide comfort when no one could visit. Many described how this felt unethical and how they had to use different strategies to cope with this challenging situation.

The fact that relatives were not present contributed to depersonalisation of the patients, all calls from relatives were handled by others such as a physician or a counsellor. This was good as it eased work burden, but all patients became just like one ‘grey mass’ and we, the staff, become neutralised in a scary way; you just shut down to manage the situation. (Participant 125)

However, nurses described how they had to adapt to this new situation, stating that care during the pandemic just had to be “good enough,” but the notion of not being able to provide the same standards of care was challenging. Furthermore, many nurses described how their professional duties changed during the pandemic, and some nurses described that they felt more like assistants.

We do not write in the patient's diary, we do not have any contact with the patient's relatives, we do not mobilise the patients. So yes, nursing care is not prioritised, it feels like we are just medical assistants who change the patient's infusions. (Participant 95)

Each day when I came to work, I had to care for patients that I did not have the competence to care for. Each day, I saw patients just lying there, naked, and fighting for their lives, without any possibility to do something about it. Each day, I felt that I am insufficient and thus have developed stress that has affected my sleep, which made me extremely tired. I have no idea how this will affect me in the long‐term and I am not even sure that I will have the strength to continue working in healthcare after this. (Participant 174)

The notion of being unable to provide nursing care or the lack of competence in caring for the severely ill ICU patients was challenging and resulted in ethical stress among the nurses.

Many new colleagues with different experiences and competencies meant a greater responsibility for me [as an ICU‐nurse]. Even if I ‘just’ had to care for two or three patients, I also had to ensure that the other patients received appropriate care and support from my colleagues. Ethical stress is when the care that you provide does not meet your own standards. (Participant 14)

#### 6.1.3 Transition to pandemic ICU care

Although the workload and burden of care increased dramatically during the pandemic, many of the respondents managed to face the situation because they helped each other. The regular ICU staff described being grateful for the support they received from colleagues from other departments, whereas nurses who had been transferred to the ICU greatly valued the support from experienced nurses and nurse assistants.

The atmosphere that arises, when staff from several different places, that never would have worked together during normal situations, solves difficult situations with critically ill ICU patients, is strange, but I have actually experienced some of my best moments as a nurse during this time. (Participant 225)

Some participants described that they had grown professionally and learned a lot, especially from each other, and that intensive care and non‐intensive care nurses would probably collaborate more in the future. Constantly working with new colleagues, the importance of teamwork skills such as good communication, co‐operation, and team leadership became evident. This required staff to be flexible and constantly adapt to working in new teams with varying levels of competence among members.

New teams are created with colleagues from other wards, with different experience and knowledge. It's unsure what competence each individual has… what you can expect and so forth. (Participant 270)

The companionship has been amazing, with a focus on finding solutions to all problems. At the same time, the work is demanding, tragical, surrealistic, and miserable. (Participant 120)

When patients recovered, especially when discharging patients from the ICU, it was a moment of joy. This was often described as victory over the disease and gave the nurses hope for other patients and the strength to carry on the work. Additionally, many emotional moments, for example, when patients were able to communicate with their relatives for the first time in maybe weeks, were described by the participating nurses.

The moment when you hold the phone and the patient speaks to a relative for the first time in weeks, after the patient has survived mechanical ventilation and been extubated, was fantastic. It gave me goose bumps and tears burned in my eyes. (Participant 82)

After seeing several patients dying, the first patient that got extubated and says: Thank you! Was it the corona virus? I answered: Yes, you bet, but now it is over. Then we both cried tears of joy. (Participant 161)

## 7 DISCUSSION

The present study describes nurses' experiences of increasing intensive care capacity and care for COVID‐19 patients during the first phase of the pandemic in Sweden. Our quantitative results suggest that regardless of previous ICU experience, only half of the respondents perceived sufficient professional support during this extreme situation, which also was described in our qualitative findings as increased ethical stress and diminished nursing care. Despite the fact that nurses who answered the survey had long experience in the profession, a large proportion (66% non‐ICU nurses and 50% ICU nurses) felt insecure in their professional practice. Hence, the COVID‐19 pandemic has impacted not only inexperienced nurses, but also specialist trained nurses. The main findings of our qualitative analysis suggest that nurses expressed their experience in three different themes: *tumbling into chaos*, *diminished nursing care*, and *transition to pandemic ICU care*.

Our results show that at the beginning of the pandemic, nurses described the situation as tumbling into chaos. As stated by the International Council of Nurses, “Nurses have always worked under intense psychological pressure, but the current pandemic is making extraordinary demands on them both physically and mentally,” which correlates well with our results.^15^ As described by Liu et al,^16^ health care providers showed a tremendous sense of responsibility and concerted efforts in alleviating patients' suffering, including working in a totally new context, physical exhaustion due to heavy workloads and protective gear, the fear of becoming infected and infecting others, and feeling powerless to handle patients' conditions. For external staff, appropriate introduction to the intensive care context and senior support to make the transition into ICU work more sustainable is essential. However, our results show that the introduction to the COVID‐19 ICU varied in both content and length and resulted in a feeling of unpreparedness. Non‐specialized intensive care nurses felt poorly trained and prepared for treating severely ill patients. The respondents felt that they had little or no impact on their working conditions, which created further stress. Hence, our findings are relevant to the ongoing discussions regarding work shifting and nursing shortage. Quantity should never be the end goal if quality is suffering, hence “more hands” are not always the best answer when competence and training are lacking. While little is known regarding the impact of work shifting in ICU and specialist nursing, the RN4Cast study by Aiken et al^17^ showed that registered nurse‐patient ratio is of huge importance regarding mortality and quality of care. The participating nurses expressed that nursing care was severely deprioritized during the pandemic. Our results show that the nurses felt that they had to act more like medical assistants with very little time or resources for nursing care. As caring for patients with COVID‐19 is a team effort, nurses have a unique role in ICUs, where 86% of patient care time comes from nurses, while only 13% comes from physicians.^18^ Nursing is more than just observable nursing activities, as suggested by Douglas et al,^19^ who showed that nurses perform about 125 activities per hour. Nursing activities are not only technical skills; nontechnical skills such as communication and decision‐making are highly needed cognitive competencies in order to analyse signs and symptoms and prevent complications and increase patient safety. Our qualitative findings suggest that, during the pandemic, several nursing tasks were deprioritized due to increased workload, as well as altered routines and a changed competence mix in the ICU, which might have affected patient outcome. Missed nursing care is a key element for patient safety.^20^ The impact of missed nursing care has been reported from both patients and nurses. Ball et al^21^ showed that each 10% increase in missed nursing care is associated with a 16% greater risk of death following surgery. While the study by Ball et al was not ICU specific, it raises significant questions about the importance of nursing care, not only for patients but also for nurse retention. One possible solution is that during extreme workload, such as throughout an ongoing pandemic, nursing care must be reorganized so that qualified bedside ICU nurses can focus on proving nursing care.

Missed nursing care has also been shown to have a great impact on nurses' turnover and burnout,^22^ which correlates with our result of nurses reporting ethical stress. Several aspects, for example, increased workload, not being able to give the normal standard of care, or lack of competence, contributed to increased ethical stress among nurses, which correlates with the results of Bambi et al^23^ and Morely et al^24^ The results show that there are three overarching ethical issues that are likely to affect nurses in unique ways: the safety of nurses, patients, colleagues, and families; the allocation of scarce resources; and the changing nature of nurses' relationships with patients and families. The fact that the hardest trial on the nurses was to offer substandard treatment and care due to lack of resources and competencies is similar to the issues raised by Bambi et al^23^ Not being able to provide the best standard of care can result in negative psychological effects such as moral stress.^25^ As ethical stress has been linked to burnout with probably intention to leave, and the shortage of registered nurses is a real problem in many countries, it is pivotal that this issue is highlighted in clinical practice and future research.

As respondents reported that COVID‐19 became the “new normal,” they expressed more positive adjustments towards the new situation with new colleagues and extreme working conditions with increased workload due to a higher nurse‐patient ratio. This correlates with the result from Sun et al,^26^ which shows nurses' growth under psychological pressure when caring for patients with COVID‐19. In line with our results, Liu et al^16^ reported that Chinese nurses and physicians were overwhelmed by their workload and experienced an uncertainty and fear of being infected by COVID‐19 and infecting others. The fact that psychological and mental health among first‐line health care workers was affected during the pandemic has been shown both in Italy and China,^9^, ^27^ which also strengthens our findings from a Swedish setting.

## 8 LIMITATIONS

Despite our intention to have as representative sample as possible, sampling through social media could mean that we reached only a small sample from our designated target group. Further, there is also a risk that our sample might be biased as those nurses who felt strongly about their experiences might have chosen to participate to a greater extent. However, recruiting participants via social media made it possible to reach a national population with different experiences during the same period, which was the intention. The timing of the data collection could have had an impact on the results of our study because the COVID‐19 pandemic spread differently throughout the nation. However, our results show that the experience of nurses throughout the nation is similar despite differences in the number of patients treated for COVID‐19. As this is a national sample, our results should be viewed from a Swedish perspective; hence, differences in ICU organizations, nurses' competencies, and nurse‐patient ratios between countries should be considered. Nevertheless, the findings of the present study share many similarities with other studies, not least regarding mental impact and ethical stress, and we believe that our results are transferable.

## 9 IMPLICATION AND RECOMMENDATIONS FOR PRACTICE

Our results show that a pandemic has a severe impact on nurses' work situations. For example, our results imply that the prerequisites for providing ICU care shifted with consequences such as an increased workload and diminished nursing care. This could affect long‐term mental health, result in increased staff turnover, and contribute further to nurse shortages. Several organizational issues need to be considered in clinical practice and future research in order to optimize the quality of nursing care. First, ICU nursing competence must be prioritized in the critical care team, especially when human resources are lacking, such as during the acute phase of a pandemic. Second, it is pivotal to have an introduction programme for each person who will work in the ICU. Third, nurses should receive both short‐ and long‐term support to identify and mitigate physical and psychological illness and burnout. Finally, nurses need to be represented among hospital managers and leaders in organizing and planning future pandemic ICU care to ensure that nursing care is prioritized.

## 10 CONCLUSIONS

The findings from the present study indicate that registered nurses working in the ICU context perceive diminished patient safety and quality of care during the COVID‐19 pandemic due to a sudden surge in capacity of ICU beds despite lack of competence. This could have a negative impact on nurses' physical and psychosocial well‐being, especially with regards to ethical stress. As specialized registered nurses are the backbone within the ICU setting, it is pivotal to further investigate COVID‐19's impact on the long‐term outcome of frontline health care workers and its impact on nursing care and patient outcomes.

## AUTHOR CONTRIBUTIONS

Ann‐Charlotte Falk was responsible for data collection and performed the quantitative data analysis. Lina Bergman performed the qualitative analysis. Lina Bergman, Ann‐Charlotte Falk, Axel Wolf, and Ing‐Marie Larsson contributed to the conception and design of the study and to writing and critically revising the manuscript. All authors read and approved the final version of the manuscript.
